# Human transposable elements in Repbase: genomic footprints from fish to humans

**DOI:** 10.1186/s13100-017-0107-y

**Published:** 2018-01-04

**Authors:** Kenji K. Kojima

**Affiliations:** 1 Genetic Information Research Institute, 465 Fairchild Drive, Suite 201, Mountain View, CA 94043 USA; 2 Department of Life Sciences, National Cheng Kung University, No. 1, Daxue Rd, East District, Tainan, 701 Taiwan

**Keywords:** Human repeat, Transposable elements, Repbase, Non-LTR retrotransposons, LTR retrotransposons, DNA transposons, SINE, *Crypton*, MER, UCON

## Abstract

Repbase is a comprehensive database of eukaryotic transposable elements (TEs) and repeat sequences, containing over 1300 human repeat sequences. Recent analyses of these repeat sequences have accumulated evidences for their contribution to human evolution through becoming functional elements, such as protein-coding regions or binding sites of transcriptional regulators. However, resolving the origins of repeat sequences is a challenge, due to their age, divergence, and degradation. Ancient repeats have been continuously classified as TEs by finding similar TEs from other organisms. Here, the most comprehensive picture of human repeat sequences is presented. The human genome contains traces of 10 clades (*L1, CR1, L2, Crack, RTE, RTEX, R4, Vingi, Tx1* and *Penelope*) of non-long terminal repeat (non-LTR) retrotransposons (long interspersed elements, LINEs), 3 types (*SINE1/7SL, SINE2/tRNA*, and *SINE3/5S*) of short interspersed elements (SINEs), 1 composite retrotransposon (*SVA*) family, 5 classes (*ERV1, ERV2, ERV3*, *Gypsy* and *DIRS*) of LTR retrotransposons, and 12 superfamilies (*Crypton*, *Ginger1*, *Harbinger*, *hAT, Helitron, Kolobok, Mariner, Merlin, MuDR, P, piggyBac* and *Transib*) of DNA transposons. These TE footprints demonstrate an evolutionary continuum of the human genome.

## Background

### Repbase and conserved noncoding elements

Repbase is now one of the most comprehensive databases of eukaryotic transposable elements and repeats [1]. Repbase started with a set of just 53 reference sequences of repeats found in the human genome [2]. As of July 1, 2017, Repbase contains 1355 human repeat sequences. Excluding 68 microsatellite representatives and 83 representative sequences of multicopy genes (72 for RNA genes and 11 for protein genes), over 1200 human repeat sequences are available.

The long history of research on human repeat sequences resulted in a complicated nomenclature. Jurka [3] reported the first 6 “medium reiterated frequency repeats” (MER) families (*MER1* to *MER6*). *MER1*, *MER3* and *MER5* are currently classified as the *hAT* superfamily of DNA transposons, and *MER2* and *MER6* are classified as the *Mariner* superfamily of DNA transposons. In contrast, *MER4* was revealed to be comprised of LTRs of endogenous retroviruses (ERVs) [1]. Right now, Repbase keeps *MER1* to *MER136*, some of which are further divided into several subfamilies. Based on sequence and structural similarities to transposable elements (TEs) reported from other organisms, other MER families have also been classified as solo-LTRs of ERVs, non-autonomous DNA transposons, short interspersed elements (SINEs), and even fragments of long interspersed elements (LINEs). Problems in classification also appear with recently reported ancient repeat sequences designated as “Eutr” (eutherian transposon), “EUTREP” (eutherian repeat), “UCON” (ultraconserved element), and “Eulor” (euteleostomi conserved low frequency repeat) [4, 5]. In general, the older the repeat is, the harder it is to classify. One reason for this pattern is the inevitable uncertainty of some ancient, highly fragmented repeats at the time of discovery and characterization.

Recent analyses of repeat sequences have accumulated evidence that repeat sequences contributed to human evolution by becoming functional elements, such as protein-coding regions and binding sites for transcriptional regulators [6, 7]. Due to the rapid amplification of nearly identical copies with the potential to be bound by transcriptional regulators, TEs are proposed to rewire regulatory networks [8–10].

Another line of evidence for the contribution of TEs comes from conserved noncoding elements (CNEs), which were characterized via the comparison of orthologous loci from diverse vertebrate genomes. CNEs at different loci sometimes show substantial similarity to one another and to some TEs [11], indicating that at least some of these CNE “families” correspond to ancient families of TEs. Xie et al. [11] reported 96 such CNE families, including those related to *MER121*, *LF-SINE*, and *AmnSINE1*. It was revealed that ancient repeats have been concentrated in regions whose sequences are well conserved [5]. However, resolving the origins of these repeat sequences is a challenge because of their age, divergence and degradation.

This article summarizes our current knowledge about the human repeat sequences that are available in Repbase. The map, showing the positions of repeats in the reference genome, the human genome sequence masked with the human repeat sequences in Repbase, and the copy number and the coverage length of each repeat family are available at http://www.girinst.org/downloads/repeatmaskedgenomes/. It is noteworthy that despite our continuous efforts, most ancient repeat sequences remain unclassified into any group of TEs (Table 1).

**Table 1:** Ancient repeat sequences not classified yet

Header	Consensus sequences
*Eutr*	*Eutr1, Eutr2, Eutr3, Eutr4, Eutr5, Eutr6, Eutr9, Eutr10, Eutr11, Eutr12, Eutr13, Eutr14, Eutr15, Eutr16, Eutr18*
*EUTREP*	*EUTREP2, EUTREP4, EUTREP5, EUTREP6, EUTREP7, EUTREP8, EUTREP11, EUTREP12, EUTREP14, EUTREP15, EUTREP16*
*MARE*	*MARE4, MARE7, MARE8, MARE9, MARE11*
*MamRep*	*MamRep564, MamRep605*
*MER*	*MER35, MER122, MER124, MER129, MER130, MER133A, MER133B, MER134, MER135*
*UCON*	*UCON1, UCON2, UCON4, UCON5, UCON6, UCON7, UCON8, UCON9, UCON10, UCON11, UCON12, UCON12A, UCON14, UCON15, UCON16, UCON17, UCON18, UCON19, UCON20, UCON21, UCON22, UCON23, UCON24, UCON25, UCON26, UCON27, UCON28, UCON28a, UCON28b, UCON28c, UCON31, UCON32, UCON33, UCON35, UCON36, UCON37, UCON38, UCON40, UCON41, UCON43, UCON44, UCON45, UCON46, UCON47, UCON48, UCON51, UCON53, UCON54, UCON56, UCON57, UCON58, UCON59, UCON60, UCON61, UCON62, UCON63, UCON64, UCON65, UCON66, UCON67, UCON68, UCON69, UCON70, UCON71, UCON72, UCON73, UCON75, UCON76, UCON77, UCON78, UCON80, UCON83, UCON84, UCON85, UCON87, UCON88, UCON89, UCON90, UCON91, UCON92, UCON93, UCON94, UCON96, UCON97, UCON98, UCON99, UCON100, UCON101, UCON102, UCON103, UCON105, UCON106*
*Eulor*	*Eulor1, Eulor2A, Eulor2B, Eulor2C, Eulor3, Eulor4, Eulor7, Eulor8, Eulor9A, Eulor9B, Eulor9C, Eulor10, Eulor11, Eulor12, Eulor12B_CM, Eulor12_CM*

### Repbase and RepeatMasker

RepeatMasker (http://www.repeatmasker.org/) and Censor [12] are the two most widely used tools for detecting repeat sequences in genomes of interest. These tools use sequence similarity to identify repeat sequences with the use of a prepared repeat library. The repeat library used by RepeatMasker is basically a repacked Repbase that is available at the Genetic Information Research Institute (GIRI) website (http://www.girinst.org/repbase). Censor is provided by GIRI itself and can use the original Repbase. The RepeatMasker edition of Repbase is released irregularly (once a year in the last 5 years), while the original Repbase is updated monthly. However, there are some minor discrepancies between Repbase and the RepeatMasker edition. These differences are caused by independent updates of repeat sequences and their annotations in both databases. These updates are seen especially for human repeats. These discrepancies include different names for the same repeats. For example, *MER97B* in Repbase is listed as *MER97b* in the RepeatMasker edition, *MER45* in Repbase is found as *MER45A* in the RepeatMasker edition, and *MER61I* in Repbase is found as *MER61-int* in the RepeatMasker edition. In some cases, the corresponding sequences may have less than 90% sequence identity due to independent sequence updates. The *MER96B* sequences in the two databases are only 89% identical. The consensus sequences of the *L1* subfamilies are divided into several pieces (“_5end,” which includes the 5’ UTR and ORF1, “_orf2,” which corresponds to ORF2, and “_3end,” which corresponds to the 3’ UTR) in the RepeatMasker edition to improve the sensitivity of detection.

This article does not aim to eliminate such discrepancies. Instead, some consensus sequences that were found only in the RepeatMasker edition previously were added to Repbase. In this article, all sequence entries are based on Repbase, but if those entries have different names in the RepeatMasker edition, these names are also shown in parentheses in the included Tables.

### TE classification in Repbase

Eukaryotic transposable elements are classified into two classes: Class I and Class II. Class I is comprised of retrotransposons, which transpose through an RNA intermediate. Class II is comprised of DNA transposons, which do not use RNA as a transposition intermediate. In other words, Class I includes all transposons that encode reverse transcriptase and their non-autonomous derivatives, while Class II includes all other autonomous transposons that lack reverse transcriptase and their non-autonomous derivatives. Another important piece of information is that the genomes of prokaryotes (bacteria and archaea) do not contain any retrotransposons.

Repbase currently classifies eukaryotic TEs into three groups: Non-LTR retrotransposons, LTR retrotransposons and DNA transposons [13] (Table 2). Non-LTR retrotransposons and LTR retrotransposons are the members of Class I TEs. To simplify the classification, some newly described groups are placed in these three groups. The “Non-LTR retrotransposons” include canonical non-LTR retrotransposons that encode apurinic-like endonuclease (APE) or/and restriction-like endonuclease (RLE), as well as *Penelope*-like elements (PLE) that encode or do not encode the GIY-YIG nuclease. These non-LTR retrotransposons share a transposition mechanism called “target-primed reverse transcription (TPRT),” in which the 3’ DNA end cleaved by the nuclease is used as a primer for reverse transcription catalyzed by the retrotransposon-encoding reverse transcriptase (RT) [14]. Non-LTR retrotransposons are classified into 32 clades. Short interspersed elements (SINEs) are classified as a group of non-LTR retrotransposons in Repbase. SINEs are composite non-autonomous retrotransposons that depend on autonomous non-LTR retrotransposons for mobilization [15, 16]. SINEs are classified into four groups based on the origins of their 5′ regions [17].

**Table 2:** TE classification in Repbase

Class	Clade/Superfamily^a^
Non-LTR retrotransposon	*Ambal,* ***CR1*** *, CRE,* ***Crack*** *, Daphne, Hero, I, Ingi, Jockey, Kiri,* ***L1*** *,* ***L2*** *, L2A, L2B, Loa, NeSL, Nimb, Outcast,* ***Penelope*** *, Proto1, Proto2, R1, R2,* ***R4*** *, RandI/Dualen, Rex1,* ***RTE*** *, RTETP,* ***RTEX*** *, Tad1,* ***Tx1*** *,* ***Vingi*** *,* ***SINE*** *(* ***SINE1/7SL*** *,* ***SINE2/tRNA*** *,* ***SINE3/5S*** *, SINEU)*
LTR retrotransposon	*BEL, Copia,* ***DIRS*** *,* ***Gypsy*** *,* ***Endogenous retrovirus*** *(* ***ERV1*** *,* ***ERV2*** *,* ***ERV3*** *, ERV4, Lentivirus)*
DNA transposon	*Academ,* ***Crypton*** *(* ***CryptonA*** *, CryptonF, CryptonI, CryptonS, CryptonV), Dada, EnSpm/CACTA,* ***Ginger1*** *, Ginger2/TDD,* ***Harbinger*** *,* ***hAT*** *,* ***Helitron*** *, IS3EU, ISL2EU,* ***Kolobok*** *,* ***Mariner/Tc1*** *,* ***Merlin*** *,* ***MuDR*** *, Novosib,* ***P*** *,* ***piggyBac*** *, Polinton, Sola (Sola1, Sola2, Sola3),* ***Transib*** *, Zator, Zisupton*

^a^Bold faces of clades/superfamilies show the presence of their traces (as repeats and/or domesticated genes) in the human genome

LTR retrotransposons are classified into five superfamilies (*Copia*, *Gypsy*, *BEL*, *DIRS* and endogenous retrovirus (*ERV*)), and the *ERV* superfamily is further subdivided into five groups (*ERV1*, *ERV2*, *ERV3*, *ERV4* and endogenous lentivirus). Except for the *DIRS* retrotransposons, these LTR retrotransposons encode DDE-transposase/integrase for the integration of cDNA, which is synthesized in the cytoplasm by the retrotransposon-encoding RT. The RT encoded by LTR retrotransposons uses tRNA as a primer for reverse transcription. The DDE-transposase/integrase of LTR retrotransposons resembles the DDE-transposase seen in DNA transposons, especially IS3, IS481, *Ginger1*, *Ginger2*, and *Polinton* [18]. *DIRS* retrotransposons, on the other hand, encode a tyrosine recombinase (YR), which is related to the YRs encoded by *Crypton* DNA transposons [19].

DNA transposons include very diverse groups of TEs. Repbase currently uses 23 superfamilies for the classification of DNA transposons. Most TE superfamilies encode DDE transposase/integrase [20], but *Crypton* and *Helitron* encode the YR and HUH nucleases, respectively [21, 22]. *Polinton* encodes a DDE transposase that is very closely related to the LTR retrotransposons, *Ginger1*, and *Ginger2*, but *Polinton* is an extremely long TE encoding DNA polymerase B and some structural proteins [18, 23]. *Polinton* was recently reported as an integrated virus designated Polintovirus, based on the identification of the coding regions for the minor and the major capsid proteins [24].

### Non-LTR retrotransposons

Only three groups of non-LTR retrotransposons are active in the human genome: *L1* (long interspersed element-1 (*LINE-1*)), *Alu* and *SVA* (*SINE-R/VNTR/Alu*). Thanks to their recent activity, these retrotransposons can be classified into many subfamilies based on sequence differences (Table 3). The classification and evolution of these groups is well described in several articles [25–28]; thus, these three groups are introduced briefly here.

**Table 3:** Non-LTR retrotransposons (LINEs, SINEs, and composites)

Class	Group/Clade	Consensus sequences
LINE	*L1*	*L1, L1HS, L1M1B_5, L1M1_5, L1M2A1_5, L1M2A_5, L1M2B_5, L1M2C_5, L1M2_5, L1M3A_5, L1M3B_5, L1M3C_5, L1M3DE_5, L1M3D_5, L1M4B, L1M6B_5end, L1M6_5end, L1M7_5end, L1MA1, L1MA10, L1MA2, L1MA3, L1MA4, L1MA4A, L1MA5, L1MA5A, L1MA6, L1MA7, L1MA8, L1MA9, L1MA9_5, L1 MB1, L1 MB2, L1 MB3, L1MB3_5, L1 MB4, L1MB4_5, L1 MB5, L1MB6_5, L1 MB7, L1 MB8, L1MC1, L1MC2, L1MC3, L1MC4, L1MC4B, L1MC4_5end, L1MC5, L1MCA_5, L1MCB_5, L1MCC_5, L1MD1, L1MD1_5, L1MD2, L1MD3, L1MDA_5, L1MDB_5, L1ME1, L1ME2, L1ME3, L1ME3A, L1ME3C_3end, L1ME3D_3end, L1ME3E_3end, L1ME3F_3end, L1ME4, L1ME4A, L1ME5, L1ME5_3end, L1MEA_5, L1MEB_5, L1MEC_5, L1MED_5, L1MEE_5, L1MEe_5end, L1MEf_5end, L1MEg_5end, L1ME_ORF2, L1P4a_5end, L1P4b_5end, L1P4c_5end, L1P4d_5end, L1P4e_5end, L1PA10, L1PA11, L1PA12, L1PA12_5, L1PA13, L1PA13_5, L1PA14, L1PA14_5, L1PA15, L1PA16, L1PA16_5, L1PA17_5, L1PA2, L1PA3, L1PA4, L1PA5, L1PA6, L1PA7, L1PA7_5, L1PA8, L1 PB1, L1 PB2, L1PB2c, L1 PB3, L1 PB4, L1PBA1_5, L1PBA_5, L1PBB_5, L1PREC1, L1PREC2, L1P_MA2, HAL1, HAL1B (HAL1b), HAL1M8, IN25, MER25, X9_LINE*
	*CR1*	*CR1L, CR1_HS, CR1_Mam, L3, L3b_3end, X1_LINE, X2_LINE, X5A_LINE, X5B_LINE, X6A_LINE, X6B_LINE, X7A_LINE, X7B_LINE, X7C_LINE, X7D_LINE, X8_LINE, X17_LINE, X18_LINE, X19_LINE, X20_LINE, X21_LINE*
	*L2*	*L2, L2B, L2C, L2D, X15_LINE, X24_LINE, UCON49, UCON86*
	*Crack*	*X13_LINE*
	*RTE*	*X3_LINE, X11_LINE, UCON82*
	*RTEX*	*L4, L5, ALINE*
	*R4*	*X4_LINE*
	*Vingi*	*X12_LINE*
	*Tx1*	*MARE6*
	*Penelope*	*UCON13*
SINE	*SINE1/7SL*	*(AluY)* *ALU, AluY, AluYa1, AluYa4, AluYa5, AluYa8, AluYb10, AluYb11, AluYb3a1, AluYb3a2, AluYb8, AluYb8a1, AluYb9, AluYbc3a, AluYc1, AluYc2, AluYc5, AluYd2, AluYd3, AluYd3a1, AluYd8, AluYe2, AluYe5, AluYf1, AluYf2, AluYf5, AluYg6, AluYh9, AluYi6, AluYk11, AluYk12, AluYk13*
		*(AluS)* *AluSc, AluSc5, AluSc8, AluSg, AluSg1, AluSg4, AluSg7, AluSp, AluSq, AluSq10, AluSq2, AluSq4, AluSx, AluSx3, AluSx4, AluSz, AluSz6*
		*(AluJ)* *AluJb, AluJo, AluJr, AluJr4*
		*(Monomeric Alu)* *FAM, FLAM, FRAM, PB1D11*
	*SINE2/tRNA*	*MIR, MIR3, MIRb, MIRc, THER1, THER2, MARE3, UCON3, UCON55, LFSINE_Vert, LmeSINE1b, LmeSINE1c, MamSINE1*
	*SINE3/5S rRNA*	*AmnSINE1_HS, DeuSINE*
	Unclassified	*MER131*
Composite	*SVA*	*SVA2, SVA_A, SVA_B, SVA_C, SVA_D, SVA_E, SVA_F*

Consensus sequences of *Alu* are further classified into reported lineages (*AluY*, *AluS*, *AluJ* and monomeric *Alu*)

*L1* is the only active autonomous non-LTR retrotransposon in the human genome. *L1* encodes two proteins called ORF1p and ORF2p. ORF1p is the structural protein, corresponding to Gag proteins in LTR retrotransposons and retroviruses. ORF2p includes domains for endonuclease and reverse transcriptase, as well as a DNA-binding CCHC zinc-finger motif. *L1* mobilizes not only its own RNA but also other RNAs that contain 3′ polyA tails. Thus, the presence of *L1* corresponds to an abundance of processed pseudogenes, which are also called retrocopies or retropseudogenes [29]. *Alu* and *SVA* transpose in a manner dependent on the *L1* transposition machinery [15, 30, 31]. *L1* is present in most mammals, but some mammals, such as megabats, have lost *L1* activity [32].

Based on their age and distribution, *L1* lineages are classified as *L1P* (primate-specific) and *L1M* (mammalian-wide). These groups are further sub-classified into various subfamilies (Table 3). *L1PA1* (*L1* and *L1HS* in Repbase correspond to this subfamily) is the only active *L1* subfamily in the human genome. During the evolution of *L1*, the 5′ and 3′ untranslated regions (UTRs) were replaced by unrelated sequences [27]. These replacements sometimes saved *L1* from restriction by KRAB-zinc finger proteins [33].

*HAL1* (half L1) is a non-autonomous derivative of *L1* and encodes only ORF1p [34]. *HAL1s* originated independently several times during the evolution of mammals [35].

The majority of *Alu* is composed of a dimer of 7SL RNA-derived sequences. Dimeric *Alu* copies in the human genome are classified into three lineages: *AluJ*, *AluS* and *AluY*, among which *AluY* is the youngest lineage [36]. Older than *AluJ* are monomeric *Alu* families, which can be classified into 4 subfamilies: *FAM*, *FLAM-A*, *FLAM-C* and *FRAM* [37]. *FLAM-A* is very similar to *PB1* from rodents; thus, Repbase does not include *FLAM-A*. *FLAM* in Repbase corresponds to *FLAM-C*. 7SL RNA-derived SINEs are called SINE1. SINE1 has been found only in euarchontoglires (also called supraprimates), which is a mammalian clade that includes primates, tree shrews, flying lemurs, rodents, and lagomorphs [38]. The close similarity between *FLAM-A* and *PB1* indicates their activity in the common ancestor of euarchontoglires, and the lack of SINE1 outside of euarchontoglires indicates that SINE1 evolved in the common ancestor of euarchontoglires after their divergence from laurasiatherians. In rodents, no dimeric *Alu* has evolved. Instead, *B1*, which is another type of derivative of *PB1*, has accumulated. The genomes of tree shrews contain composite SINEs that originated from the fusion of tRNA and 7SL RNA-derived sequences [39].

Several *Alu* subfamilies are transposition-competent. The two dominant *Alu* subfamilies that show polymorphic distributions in the human population are *AluYa5* and *AluYb8*. *AluYa5* and *AluYb8* correspond to approximately one-half and one-quarter of human *Alu* polymorphic insertions, respectively [40]. *AluYa5* and *AluYb8* have accumulated 5 and 8 nucleotide substitutions, respectively, from their ancestral *AluY*, which remains active and occupies ~15% of the polymorphic insertions. Until recently, all active *Alu* elements were believed to be *AluY* or its descendants [40]. However, a recent study revealed that some *AluS* insertions are polymorphic in the human population, indicating that some *AluS* copies are or were transposition-competent [41]. Monomeric *Alu* families are older than dimeric *Alu* families, but monomeric *Alu* families also show species-specific distributions in the great apes [37]. Monomeric *Alu* insertions have been generated via two mechanisms. One mechanism is recombination between two polyA tracts to remove the right monomer of dimeric *Alu*, and the other mechanism is the transposition of a monomeric *Alu* copy. BC200, which is a domesticated *Alu* copy [42], is the main contributor to the latter mechanism, but at least one other monomeric *Alu* copy also contributed to the generation of new monomeric *Alu* insertions [37].

*SVA* is a composite retrotransposon family, whose mobilization depends on *L1* protein activity [30, 31]. Two parts of *SVA* originated from *Alu* and *HERVK10*, which is consistent with the younger age of *SVA* than *Alu* and *HERVK10* [43]. The other parts of *SVA* are tandem repeat sequences: (CCCTCT) hexamer repeats at the 5′ terminus and a variable number of tandem repeats (VNTR) composed of copies of a 35–50 bp sequence between the *Alu*-derived region and the *HERVK10*-derived region. *SVA* is found only in humans and apes. Gibbons have three sister lineages of *SVA*, which are called *LAVA* (*L1-Alu*-VNTR-*Alu*)*, PVA* (*PTGR2*-VNTR-*Alu*) and *FVA* (*FRAM*-VNTR-*Alu*) [44, 45]. These three families share the VNTR region and the *Alu*-derived region but exhibit different compositions.

*SVA* in hominids (humans and great apes) is classified into 6 lineages (*SVA_A* to *SVA_F*), and *SVA_F* is the youngest lineage [43]. The three youngest subfamilies, *SVA_F*, *SVA_E* and *SVA_D,* contribute to all known polymorphic *SVA* insertions in the human genome. Recently, another human-specific *SVA* subfamily was found, and this subfamily has recruited the first exon of the microtubule-associated serine/threonine kinase 2 (*MAST2*) gene [46–48]. The master copy of this human-specific subfamily is presumed to be inserted in an intron of the *MAST2* gene and is transcribed in a manner dependent on *MAST2* expression in some human individuals, although it is not present in the human reference genome. An *SVA_A*-related subfamily was recently found in the Northern white-cheeked gibbon (*Nomascus leucogenys*) and was designated as *SVA*_*NLE*_ [45].

**Fig. 1:**
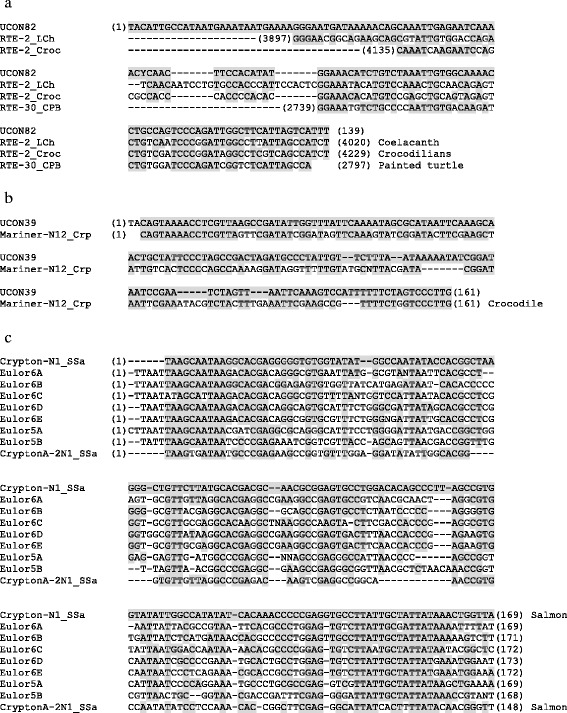
Nucleotide sequence alignments of ancient repeats with characterized TEs. Nucleotides identical to the uppermost sequence are shaded. Numbers in parentheses indicate the nucleotide position in the consensus. **a** UCON82 is an RTE non-LTR retrotransposon family. **b** UCON39 is an ancient Mariner DNA transposon family. **c** Eulor5 and Eulor6 are ancient Crypton DNA transposon families

In addition to the sequences described above, the human genome contains many signs of the ancient activity of non-LTR retrotransposons belonging to *L2, CR1*, *Crack*, *RTE*, *RTEX, R4*, *Vingi*, *Tx1* and *Penelope* (Table 3). With the rapid increase of information about repeats in other vertebrate genomes, TEs from other vertebrates occasionally provide clues about the origin of human repeat sequences. One recently classified example is *UCON82,* which exhibits similarity to the 3′ tails of vertebrate *RTE* elements from coelacanth (*RTE-2_LCh*), crocodilians (*RTE-2_Croc*) and turtle (*RTE-30_CPB*) (Fig. 1a). The characterization of *L2-3_AMi* from the American alligator *Alligator mississippiensis* revealed the *L2* non-LTR retrotransposon-like sequence signatures in *UCON49* and *UCON86*.

These groups of non-LTR retrotransposons are also found in several mammals or amniotes, supporting their past activity. *L2* is the dominant family of non-LTR retrotransposons in the platypus genome [49]. The diversification of *CR1* is a trademark of bird genomes [50]. Active *RTE* was found in various mammals and reptiles and is represented by *Bov-B* from bovines [51, 52]. *L4* and *L5* were originally classified as *RTE*, but the reanalysis revealed that these sequences are more closely related to *RTEX*. Non-LTR retrotransposons belonging to the *R4* clade were reported in the anolis lizard [53]. *Vingi* was reported in hedgehogs and reptiles [54]. Some sequence-specific non-LTR retrotransposons belonging to *Tx1* are reported in crocodilians [17]. *Crack* and *Penelope* have not been reported in any amniotes. On the other hand, *R2*, which is a non-LTR retrotransposon lineage that is distributed widely among animals [55], is not found in any mammalian genomes.

The human genome also contains many ancient SINE insertions, such as *MIRs* or *DeuSINEs* [56–58]. It is known that *MIRs* exhibit sequence similarity to *L2* in their 3′ regions, indicating that *MIRs* were transposed in a manner dependent on the transposition machinery of *L2* [49]. *MER131* is considered to be a SINE because it ends with a polyA tail. As shown in many reports [6, 59], some of these insertions have been exapted to function as promoters, enhancers or other non-coding functional DNA elements.

### LTR retrotransposons

The group of LTR retrotransposons in the human genome is primarily endogenous retroviruses (ERVs) (Table 4). *ERV1*, *ERV2* and *ERV3* are all found in the human genome, but the recently recognized *ERV4* has not been detected [60]. Neither the endogenous lentivirus nor the endogenous foamy virus (Spumavirus) was found. Some traces of *Gypsy* LTR retrotransposons have also been found, and this finding is consistent with the domesticated *Gypsy* (*Sushi*) sequences in *peg10* and related genes [61]. There are no traces of the *Copia*, *BEL* or *DIRS* retrotransposons in the human genome [62], except for the two genes encoding *DIRS*-derived protein domains: Lamin-associated protein 2 alpha isoform (LAP2alpha) and Zinc finger protein 451 (ZNF451) [63]. *BEL* and *DIRS* are found in the anolis lizard genome but have not been detected in bird genomes [62]. Mammalian genomes contain only a small fraction of *Gypsy* LTR retrotransposons, and it is speculated that during the early stage of mammalian evolution, LTR retrotransposons lost their competition with retroviruses.

**Table 4:** LTR retrotransposons and endogenous retroviruses

Superfamily	Group^a^	Internal portion	Associated LTRs
*ERV1*	*MLLV*	*HERVS71*	*LTR6A, LTR6B*
	*HERVERI*	*HERVE*	*LTR2*
		*HERVE_a*	*LTR2B, LTR2C*
		*HERV3*	*LTR4, LTR76, LTR61*
		*HERV1_I*	*HERV1_LTR, HERV1_LTRb, LTR35A*
		*HERV15I (HERV15)*	*LTR15*
		*HERVI*	*LTR10B, LTR10B1*
		*HARLEQUIN*	*HARLEQUION_LTR*
	*HERVW9*	*HERV17*	*LTR17*
		*HERV9, PTR5*	*LTR12, LTR12B, LTR12C, LTR12D, LTR12E, LTR12F*
		*HERV30I (HERV30)*	*LTR30*
		*HERV35I*	*LTR35, LTR35B*
		*MER41I (MER41-int)*	*MER41A, MER41B, MER41D, MER41E, MER41F, MER41G*
	*HERVIPADP*	*HERVIP10F, HERVIP10FH*	*LTR10F, LTR10A*
		*HERVP71A_I (HERVP71A)*	*LTR71A, LTR71B*
	*MER50like*	*MER50I (MER50-int)*	*MER50*
		*MER57I (MER57-int), MER57A_I (MER57A-int)*	*MER57A1, MER57B1, MER57B2, MER57C1, MER57C2, MER57D, MER57E1, MER57E2, MER57E3, MER57F*
		*MER84I (MER84-int)*	*MER84*
	*HERVHF*	*HERVH*	*LTR7A, LTR7B, LTR7C, LTR7Y*
		*HERVH48I (HERVH48)*	*MER48, LTR21A, MER72*
		*HERVFH19I (HERVFH19)*	*LTR19*
		*HERV19I*	*LTR19A*
		*HERVFH21I (HERVFH21)*	*LTR21B, LTR21C*
		*HERV46I (LTR46-int)*	*LTR46*
		*HERV-Fc1*	*HERV-Fc1_LTR1, HERV-Fc1_LTR2, HERV-Fc1_LTR3*
		*HERV-Fc2*	*HERV-Fc2_LTR*
		*LTR46I*	*LTR46*
	*HERVFRDlike*	*PRIMA41*	*MER41C*
		*PABL_AI*	*PABL_A*
		*PABL_BI*	*PABL_B*
		*HERV4_I, MER51I (MER51-int)*	*HERV4_LTR, MER51A, MER51B, MER51C, MER51D, MER51E, MER61D*
		*MER66_I (MER66-int)*	*MER66C*
		*HERV39 (LTR39-int)*	*LTR39*
		*?*	*PrimLTR79*
		*ERV3–1-i*	*LTR58*
	*HEPSI*	*MER65I (MER65-int)*	*MER65C, MER65A, MER65B, MER65D*
		*MER21I (MER21-int)*	*MER21, MER21A, MER21B, MER21C, MER21C_BT*
		*MER61I (MER61-int)*	*MER61C*
		*PRIMA4_I*	*PRIMA4_LTR*
		*PRIMAX_I (PRIMAX-int)*	
		*MER34-int*	*MER34*
		*MER34B_I (MER34B-int)*	*MER34B*
		*MER4I (MER4-int)*	*MER4A, MER4A1, MER4A1_LTR (MER4A1_), MER4C, MER4CL34, MER4D (MER4D0), MER4D1, MER4D_LTR, MER4E, MER4E1*
		*MER4BI (MER4B-int)*	*MER4B, LTR39*
		*MER89I (MER89-int)*	*MER89*
		*HERV38I (LTR38-int)*	*LTR38, LTR38A1, LTR38B, LTR38C*
		*MER31_I (MER31-int)*	*MER31, MER67A, MER67B, MER67C, MER67D*
		*LOR1I*	*LOR1, LOR1a_LTR, LOR1B_LTR*
		*LTR43_I (LTR43-int)*	*LTR43, LTR43B*
		*MER101_I (MER101-int)*	*MER101*
		*ERV24_Prim*	*LTR24*
		*ERV24B_Prim*	*LTR24B*
		*LTR37-int*	*LTR37A, LTR37B*
	*HUERSP*	*HUERS-P1*	*LTR8, LTR8A, LTR8B, LTR35, LTR73, LTR19B, LTR19C*
		*HUERS-P2*	*LTR1, LTR1A1, LTR1A2, LTR1B, LTR1B0, LTR1B1, LTR1C, LTR1C1, LTR1C2, LTR1C3, LTR1D, LTR1D1, LTR1E, LTR1F, LTR1F1, LTR1F2, LTR28, LTR28B, LTR28C*
		*HUERS-P3*	*LTR9A1, LTR9B, LTR9C, LTR9D, MER61A, MER61B, MER61E, MER61F*
		*HUERS-P3B*	*LTR9, LTR25*
		*MER83AI (MER83A-int)*	*MER83*
		*MER83BI (MER83B-int)*	*MER83B, MER83C*
		*HERVG25, LTR25-int*	*LTR25*
		*MER52AI (MER52-int)*	*MER52A, MER52B, MER52C, MER52D, LTR27D, LTR27E*
	Unclassified	*HERV23 (LTR23-int)*	*LTR23*
		*HERV49I (LTR49-int)*	*LTR49*
		*MER110_I (MER110-int)*	*MER110, MER110A*
	LTRs not associated with characterized internal portions		*LTR06, LTR9, LTR24C, LTR26, LTR26B, LTR26C, LTR26D, LTR26E, LTR27, LTR27B, LTR27C, LTR29, LTR31, LTR34, LTR36, LTR44, LTR45, LTR45B, LTR45C, LTR48, LTR48B, LTR51, LTR54, LTR54B, LTR56, LTR59, LTR60, LTR60B, LTR64, LTR65, LTR68, LTR70, LTR72, LTR72B, LTR75_1, LTR78, LTR78B, LTR81A, LTR81AB, LTR81B, LTR81C, LTR2752, MER31A, MER31B, MER34A, MER34A1, MER34C, MER34C2, MER34D, MER39, MER39B, MER49, MER50B, MER50C, MER66A, MER66B, MER66D, MER72B, MER87, MER87B, MER88, MER90, MER90a_LTR (MER90a), MER92A, MER92B, MER92C, MER93, MER95, MER101B*
*ERV2*	*HML1*	*HERV-K14I (HERVK14)*	*LTR14A, LTR14B*
	*HML2*	*HERVK*	*LTR5, LTR5A*
	*HML3*	*HERVK9I (HERVK9)*	*MER9a1, MER9a2, MER9a3*
	*HML4*	*HERVK13I (HERVK13)*	*LTR13, LTR13A*
	*HML5*	*HERVK22I (HERVK22)*	*LTR22A, LTR22B, LTR22B1, LTR22B2, LTR22C, LTR22C0, LTR22C2*
	*HML6*	*HERVK3I*	*LTR3, LTR3A, LTR3B*
	*HML7*	*HERVK11DI (HERVK11D)*	*MER11D*
	*HML8*	*HERVK11I (HERVK11)*	*MER11A, MER11B, MER11C*
	*HML9*	*HERV-K14CI (HERVK14C)*	*LTR14C*
	*HML10*	*HERVKC4*	*LTR14*
	LTRs not associated with characterized internal portions		*LTR5B, LTR5_Hs, LTR22, LTR22E, MER9, MER9B, RLTR10B, RLTR10C*
*ERV3*	*HERVL*	*HERVL*	*MLT2A1, MLT2A2, MLT2B3, MLT2C2, MLT2D, MLT2F*
		*ERVL*	*MLT2B2*
		*ERVL-B4*	*MLT2B4*
		*ERVL-E*	*MLT2E*
		*ERV3–16A3_I*	*ERV3–16A3_LTR, LTR16A, LTR16A1, LTR16A2, LTR16B, LTR16B1, LTR16B2, LTR16C, LTR16D, LTR16D1, LTR16D2, LTR16E, LTR16E1, LTR16E2*
		*HERV16*	*LTR16*
		*ERVL47*	*LTR47B, LTR47B2, LTR47B3, LTR47B4*
	*HERVS*	*HERV18 (HERVL18)*	*LTR18A, LTR18B, LTR18C*
		*HERVL66I (HERVL66)*	*LTR66*
	*MaLR*	*MLT-int*	*MLT1A0, MLT1A1*
		*MLT1F_I (MLT1F-int)*	*MLT1E, MLT1E1, MLT1E1A, MLT1E2, MLT1F, MLT1F1, MLT1F2*
		*MLT1H_I (MLT1H-int)*	*MLT1H*
		*MLT1J-int*	*MLT1J*
		*MLT1_I (MLT1-int)*	*MLT1C, MLT1C1, MLT1C2*
		*MST_I (MST-int)*	*MSTA, MSTA1, MSTA2 (MSTB2), MSTB, MSTB1, MSTC, MSTD*
		*THE1_I*	*THE1A, THE1B, THE1C, THE1D* *MLT1B, MLT1D, MLT1G, MLT1G1, MLT1G2, MLT1G3, MLT1H1, MLT1H2, MLT1I, MLT1J1, MLT1J2, MLT1K, MLT1L, MLT1M, MLT1N2, MLT1O*
	Unclassified	*HERVL68, MER68_I (MER68-int)*	*MER68A (MER68), MER68B, MER68C*
		*HERVL_40 (HERVL40)*	*LTR40A, LTR40A1, LTR40B, LTR40C*
		*HERVL74*	*MER74C*
		*HERV52I (LTR52-int)*	*LTR52*
		*HERV57I (LTR57-int)*	*LTR57*
		*MER70_I (MER70-int)*	*MER70A, MER70B, MER70C*
		*MER76-int*	*MER76*
		*LTR53-int*	*LTR53, LTR53B*
	(LTRs not associated with characterized internal portions)		*LTR32, LTR33, LTR33A, LTR33B, LTR33C, LTR41, LTR41B, LTR41C, LTR42, LTR47A, LTR47A2, LTR50, LTR55, LTR62, LTR67B, LTR69, LTR75, LTR75B, LTR79, LTR80A, LTR80B, LTR82A, LTR82B, LTR83, LTR84a, LTR84b, LTR86A1, LTR86A2, LTR86B1, LTR86B2, LTR86C, LTR87, LTR89, LTR91, LTR108d_Mam, LTR108e_Mam, MER54, MER54A, MER54B, MER73, MER74, MER74A, MER74B, MER77, RMER10B*
*Gypsy*		*MamGyp-int*	*MamGypLTR1a, MamGypLTR1b, MamGypLTR1c, MamGypLTR1d, MamGypLTR2, MamGypLTR2b, MamGypLTR2c*
	(LTRs not associated with characterized internal portions)		*LTR85a, LTR85b, LTR85c, LTR88a, LTR88b, LTR88c, LTR104_Mam, MamGypLTR3*
	(Internal portions not associated with characterized LTRs)		*X1_LR, X2_LR, X3_LR, X4_LR*
Unclassified	(LTRs not associated with characterized internal portions)		*LTR11, LTR77, LTR77B, LTR90A, LTR90B, MamRep1527, EUTREP10, EUTREP13*

^a^Classification is based on [64]

Historically, human ERVs have been designated with “HERV” plus one capital letter, such as K, L or S. Difficulty in classifying ERV sequences is caused by (1) the loss of internal sequences via the recombination of two LTRs and (2) the high level of recombination between different families. Different levels of sequence conservation between LTRs and the internal portions between LTRs increases this complexity. Recently, Vargiu et al. [64] systematically analyzed and classified HERVs into 39 groups. Here, the relationship between the classification reported by Vargiu et al. and the consensus sequences in Repbase is shown (Table 4). Unfortunately, it is impossible to determine all LTRs or internal sequences in Repbase using the classification system reported by Vargiu et al. [64]. Thus, in this review, 22 higher classification ranks in Vargiu et al. [64] are used, and many solo-LTRs are classified as the *ERV1*, *ERV2*, *ERV3* and *Gypsy* superfamilies. The numbers of copies for each ERV family in the human genome are available elsewhere, such as dbHERV-REs (http://herv-tfbs.com/), and thus, the abundance or the phylogenetic distribution of each family is not discussed in this review.

*ERV1* corresponds to Gammaretroviruses and Epsilonretroviruses. In the classification scheme outlined by Vargiu et al. [64], only HEPSI belongs to Espilonretrovirus. In addition, one subgroup of HEPSI, HEPSI2, may represent an independent branch from other HEPSIs and may be related to the retrovirus-derived bird gene *Ovex1* [65]. Endogenous retroviruses related to *Ovex1* were found in crocodilians [60]. Several MER families and LTR families (*MER31A, MER31B*, *MER49*, *MER65*, *MER66* (*MER66A, MER66B, MER66C, MER66D* and *MER66_I* linked with *MER66C*), *MER87*, *MER87B*, *HERV2*3, *LTR23*, *LTR37A, LTR37B*, and *LTR39*) are reported to be related to *MER4* (*MER4* group).

*ERV2* was classified into 10 subgroups by Vargiu et al. [64]. All of these subgroups belong to the lineage Betaretrovirus. No *ERV2* elements closely related to Alpharetrovirus were detected. *HERVK* is the only lineage of ERVs that has continued to replicate within humans in the past few million years [66], and this lineage exhibits polymorphic insertions in the human population [67].

*ERV3* was historically considered to be the endogenous version of Spumavirus (foamy virus); however, the recent identification of true endogenous foamy viruses (SloEFV from sloth, CoeEFV from coelacanth and *ERV1-2_DR* from zebrafish) revealed that *ERV3* and Spumavirus are independent lineages [1, 68, 69]. The *ERVL* lineage of the *ERV3* families encodes a dUTPase domain, while the *ERVS* lineage lacks dUTPase. The distribution of *ERVL*- and *ERVS*-like ERVs in amniotes indicates that at least two lineages of *ERV3* have evolved in mammalian genomes [60].

There are many recombinants between different ERV families. *HARLEQUIN* is a complex recombinant whose structure can be expressed as *LTR2-HERVE-MER57I-LTR8-MER4I-HERVI-HERVE-LTR2*. *HERVE*, *HERVIP10F*, and *HERV9* are the closest in sequence to *HARLEQUIN*, indicating that these three *ERV1* families are the components that construct *HARLEQUIN*-type recombinant ERVs. *HERVE*, *HERVIP10* and *HERV9* are classified as HERVERI, HERVIPADP and HERVW9, respectively, in Vargiu et al. [64]. Recombinants between different families or lineages makes the classification very difficult. The extremes of recombination are the recombinants between two ERVs belonging to *ERV1* and *ERV3*. Such recombination generates *ERV1*-like envelope protein-encoding *ERV3* families, although most mammalian *ERV3* families lack envelope protein genes. *HERV18* (*HERVS*) and the related *HERVL32* and *HERVL66* are such recombinants.

### DNA transposons

As shown by Pace and Feschotte [70], no families of DNA transposons are currently active in the human genome. During the history of human evolution, two superfamilies of DNA transposons, *hAT* and *Mariner*, have constituted a large fraction of the human genome (Table 5). Autonomous *hAT* families are designated as *Blackjack*, *Charlie*, *Cheshire, MER69C* (*Arthur*) and *Zaphod*. Many MER families are now classified as non-autonomous *hAT* transposons. The *Mariner* DNA transposons that contain at least a portion of a protein coding region are *Golem (Tigger3), HsMar*, *HSTC2, Kanga*, *Tigger*, and *Zombi* (*Tigger4*). Some recently characterized repeat sequence families designated with *UCON* or *X_DNA* have also been revealed to be non-autonomous members of *hAT* or *Mariner*. For example, the alignment with *Mariner-N12_Crp* from the crocodile *Crocodylus porosus* revealed that *UCON39* is a non-autonomous *Mariner* family and the first two nucleotides (TA) in the original consensus of *UCON39* are actually a TSD (Fig. 1b). The characterization of *hAT-15_CPB* from the western painted turtle *Chrysemys picta bellii* led to the classification of *Eutr7* and *Eutr8* as *hAT* DNA transposons because those sequences exhibit similarity in the termini of *hAT-15_CPB*. Based on sequence similarity and age distribution [28], it is revealed that autonomous DNA transposon families have a counterpart: non-autonomous derivative families. *MER30*, *MER30B* and *MER107* are the derivatives of *Charlie12*. *MER1A* and *MER1B* originated from *CHARLIE3*. *TIGGER7* is responsible for the mobilization of its non-autonomous derivatives, *MER44A*, *MER44B*, *MER44C* and *MER44D*.

**Table 5:** DNA transposons

Superfamily	Consensus sequences
*Crypton*	*Eulor5A, Eulor5B, Eulor6A, Eulor6B, Eulor6C, Eulor6D, Eulor6E*
*hAT*	*BLACKJACK, CHARLIE1 (Charlie1), CHARLIE1A (Charlie1a), CHARLIE1B (Charlie1b), CHARLIE2, CHARLIE2A (Charlie2a), CHARLIE2B (Charlie2b), CHARLIE3 (Charlie3), CHARLIE4 (Charlie4), CHARLIE5 (Charlie5), CHARLIE6 (Charlie6), CHARLIE7 (Charlie7), CHARLIE8 (Charlie8), CHARLIE8A (MER102A), CHARLIE9 (Charlie9), CHARLIE10 (Charlie10), Charlie11, Charlie12, Charlie13a, Charlie13b, Charlie15a, Charlie16a, Charlie17a, Charlie18a, Charlie19a, Charlie21a, Charlie22a, Charlie24, Charlie25, Charlie26a, Charlie27, Charlie28, CHESHIRE (Cheshire), CHESHIRE_A, CHESHIRE_B, EUTREP1, EUTREP3, EuthAT-1, EuthAT-2, EuthAT-2B, EuthAT-N1, Eutr7, Eutr8, Eutr17, FORDPREFECT (FordPrefect), FORDPREFECT_A (FordPrefect_a), MARE5, MER1A, MER1B, MER3, MER5A, MER5A1, MER5B, MER5C, MER5C1, MER20, MER20B, MER30, MER30B, MER33, MER45 (MER45A), MER45B, MER45C, MER45R, MER58A, MER58B, MER58C, MER58D, MER63A, MER63B, MER63C, MER63D, MER69A (Arthur1A), MER69B (Arthur1B), MER69C (Arthur1), MER80 (Charlie4a), MER80B, MER81, MER91A, MER91B, MER91C, MER94, MER94B, MER96, MER96B, MER97A (MER97a), MER97B (MER97b), MER97C (MER97c), MER97d, MER99, MER103, MER103B, MER103C, MER105, MER106 (MER106A), MER106B, MER107, MER112, MER113, MER113B, MER115, MER117, MER119, MER121, MamRep1879, MamRep1894, MamRep38, MamRep4096, MamRep488, ORSL, ORSL-2a, ORSL-2b, UCON34, UCON50, UCON52, UCON74, UCON79, UCON81, UCON95, UCON107, UCON132a, UCON132b, X7_DNA, X15_DNA, X21_DNA, X28_DNA, X31_DNA, ZAPHOD (Zaphod), Zaphod3*
*Helitron*	*Helitron1Nb_Mam, Helitron3Na_Mam*
*Kolobok*	*UCON29*
*Mariner/Tc1*	*EutTc1-N1, GOLEM (Tigger3), GOLEM_A (Tigger3a), GOLEM_B, GOLEM_C, HSMAR1, HSMAR2, HSTC2, Kanga1, Kanga1d, KANGA2_A (Kanga2_a), Kanga11a, MADE1, MARE1, MARE10, MARNA, MER2, MER2B, MER6, MER6A, MER6B, MER6C, MER8, MER28 (Tigger2a), MER44A, MER44B, MER44C, MER44D, MER46C, MER47B, MER47C, MER53, MER82, MER104, MER104A (Kanga1a), MER104B (Kanga1b), MER104C (Kanga1c), MER116, MER127, MER132, MERX, MamRep137, MamRep434, TIGGER1 (Tigger1), TIGGER2 (Tigger2), Tigger3b, Tigger3c, Tigger3d, Tigger4a, TIGGER5 (Tigger5), TIGGER5A (MER47A), TIGGER5_A, TIGGER5_B (Tigger5b), TIGGER6A (Tigger6a), TIGGER6B (Tigger6b), TIGGER7 (Tigger7), TIGGER8 (Tigger8), TIGGER9, Tigger9b, Tigger10, Tigger12, Tigger12A, Tigger13a, Tigger14a, Tigger15a, Tigger16a, Tigger16b, Tigger2b_Pri, UCON39, UCON42, UCON104, X1_DNA, X6a_DNA, X6b_DNA, X10a_DNA, X10b_DNA, X13_DNA, X25_DNA, X26_DNA, X32_DNA, X33a_DNA, ZOMBI (Tigger4), ZOMBI_A, ZOMBI_B, ZOMBI_C*
*Merlin*	*Merlin1_HS*
*MuDR*	*RICKSHA (Ricksha), RICKSHA_0 (Ricksha_0), Ricksha_a*
*piggyBac*	*LOOPER (Looper), MER75, MER75A, MER75B, MER85*
Unclassified	*MER123, MER125, MER126, MER136, DNA1_Mam, X2a_DNA, X2b_DNA, X4a_DNA, X4b_DNA, X5a_DNA, X5b_DNA, X9a_DNA, X9b_DNA, X9c_DNA, X11_DNA, X12_DNA, X17_DNA, X18_DNA, X20_DNA, X22_DNA, X23_DNA, X24_DNA, X27_DNA, X29a_DNA, X29b_DNA, X30_DNA, X34_DNA*

In addition to these two dominant superfamilies, small fractions of human repeats are classified into other DNA transposon superfamilies (Table 5). These repeats are *Crypton* (*Eulor5A, Eulor5B, Eulor6A, Eulor6B, Eulor6C, Eulor6D and Eulor6E*), *Helitron* (*Helitron1Nb_Mam* and *Helitron3Na_Mam*), *Kolobok* (*UCON29*), *Merlin* (*Merlin1-HS*), *MuDR* (*Ricksha*), and *piggyBac* (*Looper*, *MER75* and *MER85*). A striking sequence similarity was found between *Crypton* elements from salmon (*Crypton-N1_SSa* and *CryptonA-N2_SSa*) and *Eulor5A/B* and *Eulor6A/B/C/D/E*, especially at the termini (Fig. 1c). They are the first Eulor families classified into a specific family of TEs and also the first finding of traces of *Cryptons* in the human genome, except for the 6 genes derived from *Cryptons* [71].

Like *Crypton*-derived genes, some human genes exhibit sequence similarity to DNA transposons, which have not been characterized in the human genome. The identification of these “domesticated” genes reveals that some DNA transposons inhabited the human genome in the past. Ancient *Transib* was likely the origin of the *rag1* and *rag2* genes that are responsible for V(D)J recombination [72–74]. THAP9 has a transposase signature from a *P* element and retains transposase activity [75]. *harbi1* is a domesticated *Harbinger* gene [76]. *rag1*, *rag2* and *harbi1* are conserved in all jawed vertebrates. *Gin-1* and *gin-2* show similarity to *Gypsy* LTR retrotransposons, as well as *Ginger2* DNA transposons, but are the most similar to some *Ginger1* DNA transposons from *Hydra magnipapillata* [18]. Therefore, although the traces of 4 superfamilies of DNA transposons (*Transib*, *P*, *Harbinger*, and *Ginger1*) have not found as repetitive sequences in the human genome, they have contributed to human genome evolution by serving protein-coding sequences.

### Genomic traces of human evolution

Several families of TEs are still active in the human population. *L1PA1, SVA* and several *AluY* subfamilies show polymorphism in the human population, indicating their recent activity [40, 77]. Another type of evidence for the current activity of these TEs are the somatic insertions seen in brains and cancer cells [78, 79]. *HERVK* is the only lineage of ERVs exhibiting polymorphic insertions in the human population [67].

On the other hand, human repeats have accumulated during the whole history of human evolution. These repeats are certainly not restricted to the human genome but are shared with the genomes of many other mammals, amniotes, and vertebrates. Almost all TE families are shared between humans and chimpanzees. An exception is the endogenous retrovirus family *PtERV1*, which is present in the genomes of chimpanzees and gorillas but not humans [80]. The human TRIM5alpha can prevent infection by *PtERV1*, and this can be the reason why *PtERV1* is absent in the human genome [81]. Sometimes, TE families that ceased transposition long ago in the human lineage have been active to mobilize in another lineage. The *Crypton* superfamily of DNA transposons were active in the common ancestor of jawed vertebrates, judging from the distribution of orthologous *Crypton*-derived genes [71]. *Eulor5A/B* and *Eulor6A/B/C/D/E* are shared among euteleostomi including mammals to teleost fishes and show similarity to two non-autonomous *Crypton* DNA transposons from salmon (Fig. 1c). Copies of *Crypton-N1_SSa* are over 94% identical to their consensus sequence, and copies of *CryptonA-N2_SSa* are around 90% identical to their consensus sequence. The autonomous counterpart of these two salmon *Crypton* DNA transposons may be the direct descendants of the ancient *Crypton* DNA transposon that gave birth to *Eulor5A/B* and *Eulor6A/B/C/D/E*. *UCON39* is conserved among mammals and shows similarity to the crocodilian DNA transposon family *Mariner-N12_Crp* (Fig. 1b). The distribution of these two families indicates that they are the sister lineages sharing the common ancestor. Copies of *Mariner-N12_Crp* are only around 82% identical to their consensus. Considering the low substitution rate in the crocodilian lineage, *Mariner-N12_Crp* also ceased to transpose a very long ago. These examples clarify the contribution of TEs to the human genome components. They also highlight the importance of characterizing TE sequences from non-human animals in understanding the human genome evolution.

As represented by names such as EUTREP (eutherian repeat) or Eulor (euteleostomi conserved low frequency repeat), different repeat families are shared at different levels of vertebrate groups. Jurka et al. [5] reported 136 human repeat families that are not present in the chicken genome and 130 human repeat sequences that are also present in the chicken genome. These two sets of families likely represent ancient TE families that expanded in the common ancestor of mammals and ancient TE families that expanded in the common ancestor of amniotes, respectively. Based on the carrier subpopulation (CASP) hypothesis we proposed, these TE insertions were fixed by genetic drift after population subdivision [82]. These insertions may have resulted in reduced fitness of the host organism, but it can allow the organism to escape from evolutionary stasis [83]. Once TE insertions were fixed, mutations should have accumulated to increase fitness. Increasing fitness is usually through the elimination of TE activity and the removal of TE insertions. However, some TE insertions have acquired function beneficial to the host. Indeed, ancient repeats have been concentrated in regions whose sequences are well conserved [5]. They are expected to have been exapted to have biological functions as enhancers, promoters, or insulators.

More direct evidence for the ancient transposition of TEs is seen in domesticated genes. *rag1*, *rag2*, *harbi1*, and *pgbd5* (*piggyBac*-derived gene 5) are conserved in jawed vertebrates. The most ancient gene that originated from a certain TE superfamily is a *Crypton* seen in the *woc/zmym* genes [71]. Four genes, *zmym2*, *zmym3*, *zmym4* and *qrich1,* were duplicated by two rounds of whole genome duplication in the common ancestor of vertebrates and represent the orthologs of *woc* distributed in bilaterian animals. Unfortunately, this level of conservation is unlikely to be present in non-coding sequences derived from TEs; however, over 6500 sequences are reported to be conserved among chordates, hemichordates and echinoderms [84]. Researchers are more likely to find traces of ancient TEs when analyzing slowly evolving genomes, such as crocodilians [85].

## Conclusions

Nearly all repeat sequences in the human genome have likely been detected. The current challenge is the characterization of these repeat sequences and their evolutionary history. This characterization is one objective of the continuous expansion of Repbase. Repbase will continue to collect repeat sequences from various eukaryotic genomes, which will help to uncover the evolutionary history of the human genome.

## Abbreviations

APE: Apurinic-like endonuclease
CNE: Conserved noncoding element
ERV: Endogenous retrovirus
Eulor: Euteleostomi conserved low frequency repeat
Eutr: Eutherian transposon
EUTREP: Eutherian repeat
HAL1: Half L1
L1: Long-interspersed-element-1
LINE: Long interspersed element
LTR: Long terminal repeat
MAST2: Microtubule-associated serine/threonine kinase 2.
MER: Medium reiterated frequency repeats
ORF: Open reading frame
PLE: *Penelope*-like element
RLE: Restriction-like endonuclease
RT: Reverse transcriptase
SINE: Short interspersed element
SVA: SINE-R/VNTR/Alu
TE: Transposable element
TPRT: Target-primed reverse transcription
UCON: Ultraconserved element
UTR: Untranslated regions
VNTR: Variable number of tandem repeats
YR: Tyrosine recombinase
